# Exploring Therapeutic Digestive Enzyme Landscape in India: Current Evidence, Profit Motives, Regulations, and Future Perspectives

**DOI:** 10.7759/cureus.52891

**Published:** 2024-01-24

**Authors:** Madhusudan P Singh, Nikunj R Agrawal, Sushant Saurabh, Ekta Krishna, Juhi M Singh

**Affiliations:** 1 Pharmacology and Therapeutics, All India Institute of Medical Sciences, Raipur, Raipur, IND; 2 Gastroenterology, Apollo Hospitals, Kolkata, IND; 3 Community and Family Medicine, All India Institute of Medical Sciences Patna, Patna, IND; 4 Pathology, K.D. Medical College, Mathura, IND

**Keywords:** pancreatic insufficiency, celiac disease, rational drug prescription, india, therapeutic digestive enzymes

## Abstract

This analysis critically examines the profit-driven marketing of digestive enzymes as over-the-counter (OTC) supplements in the context of India, expressing ethical concerns regarding pharmaceutical companies prioritizing financial gain over genuine public health needs within the lucrative OTC supplement market. The review delves into various enzymes, their mechanisms of action, uses, adverse drug reactions, and provides evidence from various studies. The research method involves the exploration of profit-driven strategies employed by pharmaceutical companies, addressing regulatory challenges, investigating the gap between dietary supplements and pharmaceutical drugs, and emphasizing the impact of direct-to-consumer advertising on self-diagnosis and overuse. Additionally, the study reviews various e-pharmacy platforms in India, assessing formulations and pricing. Key findings highlight the diverse formulations on these platforms, exposing insights into cost variations and indicating a regulatory gap that necessitates a comprehensive re-evaluation by Indian and international authorities. The analysis emphasizes the influence of direct-to-consumer advertising on behavior and potential health risks, raising ethical concerns about oversimplified health claims that overlook the necessity for individualized treatment plans. In conclusion, the study underscores the ethical complexity of prioritizing profit over public health and advocates for regulatory re-evaluation, exploring broader implications such as cultural influences and alternative therapies. The evolving landscape, featuring plant-based and microbe-derived alternatives, is presented as transformative, particularly in conditions like celiac disease.

## Introduction and background

Therapeutic digestive enzymes are specialized proteins that play a crucial role in facilitating the breakdown of food into absorbable nutrients within the digestive system. These enzymes are naturally produced by the body, primarily in the pancreas, and assist in the digestion of carbohydrates, proteins, and fats. However, therapeutic digestive enzymes can also be obtained from external sources, often in the form of supplements, to support individuals with digestive disorders or deficiencies.

At the heart of the promotion of unnecessary digestive enzymes lies the profit motive, with pharmaceutical companies, including those in India, capitalizing on the lucrative market of over-the-counter (OTC) supplements. The pervasive mass marketing often lacks nuance, prompting questions about prioritizing profits over genuine public health needs. This commercial interest may contribute to unwarranted consumption, thereby raising ethical concerns regarding the equilibrium between profits and the authentic health requirements of the public [1-3].

Adding to the complexity are regulatory challenges posed by government regulatory authorities in India and globally in the landscape of dietary supplements, including digestive enzymes. Unlike pharmaceutical drugs, dietary supplements face less stringent regulations, both domestically and internationally, enabling marketing without robust scientific evidence. This regulatory gap exposes consumers to products with unverified claims and potential health risks, emphasizing the pressing need for a thorough re-evaluation of the regulatory framework by Indian and international authorities alike [4,5].

Direct-to-consumer advertising further complicates the scenario, wielding influence over individuals to self-diagnose and self-prescribe digestive enzyme supplements without consulting healthcare professionals. This practice can lead to overuse without a clear understanding of necessity, potentially resulting in adverse health outcomes [1-3].

Moreover, some companies make generalized health claims about the benefits of digestive enzymes for overall well-being. This oversimplification neglects proper diagnosis and individualized treatment plans, potentially swaying consumers with optimistic promises without considering the nuances of their health needs [6].

Given these concerns, consumers are urged to approach the use of digestive enzyme supplements with caution. Seeking advice from healthcare professionals, relying on evidence-based practices, being aware of red flags in product claims, and advocating for responsible marketing practices are crucial steps in navigating this landscape. The ethical dilemmas associated with the marketing of digestive enzymes unfold against a backdrop where profit-driven strategies, including those employed by Indian pharmaceutical companies, intersect with public health, emphasizing the urgency for a comprehensive reassessment of existing regulatory frameworks. Direct-to-consumer advertising amplifies the potential for self-diagnosis, raising concerns about overuse and adverse health consequences. In response to these concerns, consumers are urged to exercise caution [1-3]. Seeking guidance from healthcare professionals, relying on evidence-based practices, discerning red flags in product claims, and advocating for responsible marketing practices become imperative steps in navigating this intricate terrain where financial interests and public health converge. The healthcare landscape is intricately woven with the progress of medical science and the strategic marketing employed by pharmaceutical companies, including those in India. This article delves into the ethical implications of marketing digestive enzymes as OTC supplements, shedding light on profit motives, regulatory challenges, direct-to-consumer advertising, and broad health claims.

## Review

The use of digestive enzymes and considerations for consumers

The intricate role of digestive enzymes in the digestive process is crucial for optimal nutrient absorption. These bioactive molecules, naturally secreted by salivary glands, stomach, pancreas, and small intestine, break down complex nutrients into simpler forms. The enzymes highlighted in this context, fungal diastase and pepsin, represent amylase and protease enzymes, respectively, playing essential roles in carbohydrate and protein digestion [7].

Deficiencies in digestive enzyme production can lead to various digestive issues such as bloating, gas, or indigestion. Factors contributing to these deficiencies include medical conditions, the aging process, or lifestyle choices. In such cases, supplementing with digestive enzymes, tailored to individual needs, can enhance the digestive process and alleviate associated symptoms [8].

Indications for digestive enzyme supplementation are diverse, ranging from pancreatic insufficiency to food intolerances like lactose or gluten intolerance. Additionally, aging is associated with a natural decline in digestive enzyme production, making enzyme supplementation beneficial for the elderly population [9]. Individuals with specific digestive disorders, including irritable bowel syndrome (IBS) or inflammatory bowel diseases (IBD), may also experience impaired digestive enzyme activity, warranting consideration for enzyme supplementation in their comprehensive management [10].

The decision to use digestive enzymes should be individualized. While these supplements can play a valuable role in promoting optimal digestion and nutrient absorption, their necessity should be determined based on careful consideration of individual health circumstances and symptoms. Consulting with a healthcare professional is crucial to assess the appropriateness of enzyme supplementation and address any underlying digestive issues.

Challenges and innovations in enzyme therapy

Enzymes, critical as therapeutic agents, face challenges in oral administration due to the dynamic environment of the gastrointestinal (GI) tract. The varying pH levels and enzymatic activities in different segments of the GI tract pose obstacles to maintaining enzyme stability. For instance, the low pH in the stomach can potentially inactivate therapeutic enzymes like pepsin, prompting the exploration of innovative strategies such as recombinant technologies and gastro-resistant coatings to enhance stability [11,12].

Challenges persist as enzymes progress through the stomach to the small intestine, where neutral pH and the presence of pancreatic peptidases and bile salts introduce additional hurdles. Various approaches, including covalent conjugation to polymers and enteric-coated formulations, are being explored to enhance enzyme stability in these environments [11,12].

Exceptional cases, such as sacrosidase used for sucrose-isomaltase intolerance, highlight enzymes that demonstrate resistance to acidic environments, possibly due to enzyme glycosylation and high concentrations. Modifications like chemical conjugation with branched polyethylene glycol (PEG) have shown improved stability in lactase supplementation for lactose intolerance [9].

Digestive enzyme supplementation in gastrointestinal disorders

Recent research highlights the expanding role of digestive enzyme supplementation in managing GI disorders, exploring plant-based and microbe-derived enzymes for therapeutic advancements [10]. The diverse landscape of available formulations, varying in enzyme type, source, origin, and dosage, emphasizes the need for careful selection of digestive enzyme supplements. Continuous research and innovation are crucial for overcoming current limitations and revolutionizing the treatment of various disorders through oral enzyme (OE) therapies.

In a two-month, double-blind, placebo-controlled trial with 120 participants aged 18-59, a fungal fermentation-derived multienzyme blend effectively alleviated functional dyspepsia symptoms, improving quality of life, pain severity, and sleep quality. This suggests a promising therapeutic approach for managing functional dyspepsia [13]. Functional dyspepsia, presenting therapeutic challenges, was addressed in a randomized, double-blind, placebo-controlled study evaluating DigeZyme®, a proprietary multienzyme complex (MEC). Over a 60-day intervention, MEC demonstrated statistically significant improvements in various dyspepsia assessment scales compared to placebo, confirming its safety and effectiveness for managing functional dyspepsia symptoms [14]. A study by the University of Catanzaro IBD Unit investigated a blend of beta-glucan, inositol, and digestive enzymes in 43 IBD patients with concurrent IBS symptoms. Group A, receiving the supplement alongside standard treatment, reported significant reductions in abdominal pain, bloating, and flatulence, highlighting the effective contribution of beta-glucan, inositol, and digestive enzyme supplementation to the enhanced clinical condition of IBD-IBS patients [15].

Pancreatic Enzyme Supplementation

Pancreatic enzyme supplementation is a preferred therapeutic approach for managing exocrine pancreatic insufficiency (EPI) associated with conditions like chronic pancreatitis, pancreatic cancer, cystic fibrosis, and diabetes [16]. EPI results from insufficient digestive enzyme production by the pancreas, affecting protein, carbohydrate, and fat digestion. Commercial formulations, primarily porcine or bovine-derived, are available, with recent advancements exploring microbe-derived enzymes, offering advantages such as lower dosage requirements and a broader pH range of activity [17]. Enteric-coated formulations protect enzymes from degradation in the acidic stomach environment, ensuring the efficacy of enzyme supplementation by protecting the enzymes from degradation [10]. Regulatory changes, including the FDA's requirement for clinical trials and Investigational New Drug Application submission since April 2010, have influenced the availability of pancreatic enzyme preparations in the United States [18]. Table 1 depicts some of the enzyme formulations available as OTC drugs in India.

**Table 1:** Various exogenous pancreatic enzyme and lactase formulations available in India* *Ref: Various online e-pharmacy & medical store platforms in India (e.g., 1mg, PharmEasy, Netmeds, etc.)

S. N.	Brand Name	Enteric Coated (Y/N)	Formulation	Lipase (Unit)	Amylase (Unit)	Protease (Unit)	Lactase (Unit)	Price Per Unit In Rupees
Exogenous Pancreatic Enzymes								
1	Creon 40000 400 mg	Y	Capsule	40000	25000	16000	-	128.25
2	Panstal plus 170 mg	Y	Capsule	10000	8000	600	-	16.05
3	Enzar Forte (170 mg/65 mg) Liquid 150 mL	-	-	-	-	-	-	85
4	Creopase 150 mg	Y	Capsule	10000	33200	37500	-	60.2
5	Panlipase 25000 300 mg	Y	Capsule	25000	74700	62500	-	
6	Enzabil 212.5 mg	Y	Tablet	6000	4500	300	-	7.85
7	Enzoxy max 150 mg	Y	Capsule	600	8000	10000	-	32.9
8	Digecran 25000 300 mg	Y	Capsule	62500	74700	25000	-	46.5
9	Enzase 20 20 mg	Y	Tablet	20000	62250	93750	-	27
10	Creon SD granules 150 mg	Y	Granules	10000	8000	600	-	363
Lactase Enzymes								
1	Cry NO 10 mL		Drops				600 IU	99
2	Surgolact		Drops				5000 FCC	191
3	Yamoo		Tablet				4500 FCC	21.9
4	Bioven 300 mg		Capsule				9000 FCC	60
5	Lactaneed 300 mg		Capsule				90000 FCC	12.5

Indications for supplementation include EPI-related symptoms like weight loss, increased fecal fat excretion, and steatorrhea, with choices between porcine, bovine, or microbial sources depending on availability and patient preferences [19]. Dosages vary based on the underlying cause, patient characteristics, and formulation type [20]. In a crossover trial, pancrealipase (PEZ) showed efficacy in reducing postprandial irritable bowel syndrome-diarrhea (IBS-D) symptoms, with 61% preference for PEZ over placebo, suggesting its potential in managing postprandial IBS-D symptoms [21].

Fungal diastase, derived from Aspergillus oryzae, aids carbohydrate digestion in pancreatic insufficiency, enhancing nutrient absorption, but may cause mild adverse effects in some individuals [22]. Pancreatic enzyme supplementation remains a crucial therapeutic strategy, with formulation choice tailored to patient needs and conditions.

Lactase (β-Galactosidase) Supplementation

Lactase deficiency, the primary cause of lactose malabsorption, underscores the importance of lactase supplementation in managing lactose intolerance. Lactase, an enzyme produced by intestinal villi, plays a crucial role in hydrolyzing lactose into its component sugars, galactose, and glucose. Primary lactose malabsorption occurs post-weaning due to a decline in lactase activity, while secondary hypolactasia can result from mucosal damage or increased GI transit time [23].

Exogenous lactase, sourced from yeast or fungi, provides a reliable therapeutic option for lactase supplementation in formulations like capsules and tablets, effectively alleviating lactose intolerance symptoms. Comparative studies have revealed variations in efficacy based on the microorganism source, emphasizing the importance of selecting appropriate lactase brands tailored to individual needs [10]. Enzymes derived from different microorganisms exhibit varying efficacy in lactose hydrolysis. Comparative studies emphasize the superior efficacy of lactase derived from Kluyveromyces lactis compared to Aspergillus niger [24,25]. Dosages recommended for lactase supplementation vary among brands, and studies demonstrate the relative equivalency of chewable, caplet, and soft-gel beta-gal products [26].

Lactase (β-galactosidase) supplementation, a key component of digestive enzyme therapy, addresses lactose intolerance resulting from the genetic basis for lactase deficiency. While guidelines do not comprehensively cover its use in lactose intolerance, enzymes sourced from yeast or fungi offer a dependable therapeutic option. Comparative studies highlighting variations in efficacy underscore the importance of considering the source of exogenous lactase when choosing supplementation [27].

Recommended dosages, contingent on enzymatic activity, allow individuals with lactose intolerance to tailor their treatment. The availability of various formulations, including capsules and tablets, provides choices to accommodate individual preferences and needs. Overall, lactase supplementation is a valuable strategy in managing lactose intolerance, contributing to improved digestive health and the overall well-being of affected individuals.

Pepsin Supplementation

Pepsin, an enzyme produced in the stomach, is essential for breaking down proteins into smaller peptides during digestion. When administered as a medicine in the form of syrup or capsules, synthetic pepsin supplements can support the digestive process. These supplements are designed to aid individuals with insufficient natural pepsin production or those experiencing difficulty digesting protein-rich foods. By facilitating the breakdown of proteins into more digestible fragments, pepsin supplements can enhance overall protein digestion and nutrient absorption. They are often recommended for conditions such as protein malabsorption or digestive disorders where there is a deficiency in endogenous pepsin (Table 2).

**Table 2:** Various digestive enzyme formulations available in India* *Ref: Various online e-pharmacy and medical store platforms in India (e.g., 1mg, PharmEasy, Netmeds, etc.)

S. N.	Brand name	Formulation	Alpha-amylase (mg)	Pepsin (mg)	Alpha-galactosidase (mg)	Lipase (mg)	Simethicone (mg)	Protease (mg)	Lactase (mg)	Diastase (mg)	Price (Rupees Per Capsule OR Rupees Per 5 mL)*	Remarks
1	Byzyme (18.75 mg/12.5 mg)	Capsule	18.75	12.5	-	-	-	-	-	-	6.190	-
2	Celcius Dizest	Capsule	50	-	150	3	-	25	7	-	9.800	-
3	Wegazyme	Syrup	-	10	-	-	60	-	-	50	2.475	Quantities in milligrams per 15 mL of syrup
4	Lupizyme SF	Syrup	18.75	12.5	-	-	-	-	-	-	3.125	Quantities in milligrams per 5 mL of syrup
5	Cadzyme	Capsule	18.75	12.5	-	-	-	-	-	-	6.200	-
6	Vivozyme	Syrup		10	-	-	50	-	-	50	3.225	Quantities in milligrams per 5 mL of syrup
7	Nutrozyme Plus	Syrup	18.75	12.5	-	-	-	-	-	-	3.125	Quantities in milligrams per 5 mL of syrup
8	Neo Enzomagic	Capsule	18.75	12.5	-	-	-	-	-	-	7.000	-
9	Watazym 5	Capsule	50	-	150	3	-	25	7	-	10.990	-

α-Glucosidase

α-Glucosidase, an enzyme derived from various sources such as animals, plants, bacteria, and fungi, plays a crucial role in digestion by breaking down di- and oligosaccharides, as well as aryl glucosides, to yield glucose. In the human digestive system, this enzyme facilitates the hydrolysis of complex carbohydrates, including maltooligosaccharides and soluble starch, into simpler sugars like glucose. Plants naturally contain α-glucosidase as an endocellular enzyme, influencing the composition of plant polysaccharides during maturation. Specific sources of α-glucosidase, such as corn and rice, exhibit substrate preferences, with corn α-glucosidase producing glucose exclusively from starch. Additionally, α-glucosidase can collaborate with other enzymes, such as alpha-amylase, to enhance the rate of starch hydrolysis. The enzyme is also produced by bacteria like *Bacillus subtilis* and *Thermus thermophilus*, contributing to the digestion of maltooligosaccharides and starch. Fungi, including *Mucor javanicus* and *Lentinus edodes*, produce α-glucosidase with glucosyltransferase activity, further emphasizing its significance in carbohydrate digestion [28].

Enzyme supplementation in celiac disease

Celiac disease (CD) is a chronic autoimmune disorder triggered by gluten ingestion, causing inflammation and small intestine damage. The standard CD management involves a strict gluten-free diet, excluding wheat, barley, and rye. Recent research focuses on enzyme supplementation, specifically prolyl endopeptidases (PEPs). PEPs, categorized as serine proteases, uniquely target proline-rich regions in gluten protein structure, showing promise in breaking down gluten molecules in both in-vitro and in-vivo studies. This potential therapy extends to combination enzyme therapy, complementing the limitations of a gluten-free diet, especially considering challenges in dietary adherence and hidden gluten sources.

A placebo-controlled trial assessed enzyme therapy's efficacy in 21 CD patients undergoing a two-week gluten challenge. Compared to the placebo, enzyme therapy demonstrated improved symptom scores (p<0.02), with 38% experiencing over five moderate-severe symptoms during challenges. Enzyme therapy also provided superior mucosal protection during gluten challenges, suggesting its potential as a supplement for CD patients on a strict gluten-free diet [29].

Despite promising insights, commercial PEP preparations for CD treatment are not yet available. Further exploration through larger clinical trials is essential to establish the safety, efficacy, and optimal dosage of PEPs in real-world settings. Enzyme supplementation, particularly with PEPs, offers a potential avenue for advancing CD management, providing new possibilities beyond the constraints of a gluten-free diet. While current evidence is promising, incorporating PEPs into mainstream CD treatment requires rigorous research and validation through larger clinical studies, offering hope for improved outcomes and enhanced quality of life for affected individuals. Various enzyme formulations commercially available in India are depicted in Tables 1-4.

**Table 3:** Various digestive enzyme + prebiotics/probiotics combination formulations available in India* *Ref: Various online e-pharmacy and medical store platforms in India (e.g., 1mg, PharmEasy, Netmeds, etc.)

Brand	Preparation	Enzyme	Probiotic	MRP (in Rupees Per Capsule)	Remark
Vitazyme	Capsule	Diastase	Lactic acid bacillus	8	10 cap
Unienzyme Pro	Capsule	Alpha-amylase	Saccharomyces boulardii	15.9	10 cap
		Alpha-galactosidase	Lactobacillus sporogenes		
		papain	Staphylococci, *Saccharomyces boulardii*		
		Lactase	Lactobacillus sporogenes		
		Glucoamylase	Streptococcus faecalis		
		Invertase	Clostridium botulinum		
		Protease	Bacillus mesentericus		
		Cellulase	Fructooligosaccharides		
		Beta glucanase			
NeuRapid Pre Probiotics	Capsule	Amylase	*Lactobacillus* species	10.81	60 cap
		Maltase			
		Lipase			
		Protease			
		Cellulase			
		Papain			
		Lactase			

**Table 4:** Other miscellaneous combinations of digestive enzyme formulations available in India* *Ref: Various online e-pharmacy and medical store platforms in India (e.g., 1mg, PharmEasy, Netmeds, etc.)

Brand	Preparation	Enzyme	Miscellaneous add-ons	MRP (in Rupees Per Capsule)	Remark
Zymetral	Syrup	Fungal Diastase	Niacinamide	88.75	200 mL
		Pepsin	Thiamine		
			Pyridoxine		
			Riboflavin		
			Vitamin B 12		
Anzyme	Syrup	Fungal Diastase	Papain	102	200 mL
			Activated charcoal		
Xymex MPS	Tablet	Fungal Diastase	Papain	45	10 tablets
			Simethicone		
Vitazyme drops	Drops	Fungal Diastase	Cinnamon oil, caraway oil, cardamom oil	92	15 mL
Pankreoflat	Tablet	Pancreatin	Dimethicone	102	10 tablets

Rational design of enzyme combination therapy

The evolving landscape of digestive enzyme supplementation introduces the concept of a “super-enzyme” formulation, featuring a diverse array of enzymes with potential applications in severe pancreatic insufficiency, malabsorption syndromes, severe malnutrition, and specific demographics like the elderly or infants. Theoretical formulations may strategically combine enzymes, addressing issues like impaired bicarbonate secretion in pancreatic insufficiency through the inclusion of a proton pump inhibitor (PPI). Integration of conjugated bile acids with pancreatic enzymes is explored, particularly in cases with biliary etiology.

Innovatively, the addition of probiotics to enzyme supplementation offers a novel therapeutic dimension. Tailoring probiotics to specific digestive disorders, such as lactose intolerance or pancreatic insufficiency, is a promising approach. Potential applications include combining digestive enzymes and probiotics for conditions like CD, with A. niger, a source of endopeptidases, considered for inclusion [10,25]. Despite promising hypotheses, further research is essential to establish the efficacy of these combinations and potentially integrate them into clinical practice.

Future perspectives in enzyme replacement therapy

Enzyme replacement therapy is evolving beyond its traditional role in addressing enzyme deficiency, extending its reach to conditions like CD. The fusion of different enzymes is being explored for tailored therapeutic options, showcasing an evolving understanding of enzyme functions. In the realm of future perspectives, probiotic supplementation tailored to specific digestive disorders emerges as a promising avenue. The potential synergies between digestive enzymes and probiotics offer a holistic approach to digestive health, redefining the treatment paradigm for digestive disorders.

Therapeutic use of enzymes is also explored in neoplasms or end-stage diseases. In a retrospective cohort of 1,242 colorectal cancer patients, postoperative OE treatment complementing anti-neoplastic therapy showed a significant reduction in symptoms and decreased adverse reactions [30]. A similar positive impact was observed in a retrospective cohort analysis of 2,339 breast cancer patients, where OE enhanced quality of life and minimized symptoms and therapy side effects [31].

In a double-blind, randomized clinical trial involving 101 children with autism spectrum disorders (ASDs), a three-month digestive enzyme therapy demonstrated significant improvements in various ASD symptoms compared to a placebo. Despite these promising findings, further investigation into long-term effects and optimal dosage is warranted [32].

Amid these advancements, the challenges and successes in OE therapy remain pivotal, emphasizing the imperative for continuous research and innovation. The pursuit of improved stability through polymer conjugation and protein engineering stands as a beacon of promise for enhancing the effectiveness of OE therapies. Furthermore, the targeted addressal of disease-specific challenges, such as ensuring stability in acidic conditions or resistance to digestive enzymes, remains a focal point for future developments. These endeavors aim not only to overcome current limitations but also to set the stage for the efficacious treatment of a spectrum of conditions through the evolving landscape of OE therapies.

Indian scenario

The landscape of digestive enzyme supplements in India reveals not only ethical concerns but also a concerning disparity in pricing among similar products, underscoring the profit-driven strategies adopted by various pharmaceutical companies. The same digestive enzyme product is often priced differently by different companies, creating a substantial gap in pricing that raises questions about transparency and fair market practices. Moreover, the pricing inconsistencies are compounded by the alarming revelation that many of these products, along with their varied strengths, are not approved by the Central Drugs Standard Control Organization (CDSCO), the government regulatory authority in India. This regulatory gap not only exposes consumers to potential health risks but also emphasizes the need for stringent oversight to ensure the safety and efficacy of these supplements. The wide range of pricing coupled with the lack of regulatory approval by the CDSCO unveils a complex scenario where profit motives and regulatory oversights intersect, further deepening the ethical and health-related concerns associated with the marketing of digestive enzyme supplements in India (Tables 1-4). Regulatory bodies should collaborate with experts in the field to establish clear and updated guidelines for the production, labeling, and marketing of therapeutic digestive enzymes. These guidelines should encompass quality control measures, safety assessments, and standardized testing protocols to ensure the efficacy and safety of these products. Regular audits and inspections of manufacturing facilities can help enforce compliance. Strengthening the enforcement of existing regulations and imposing stricter penalties for violations can act as a deterrent. Law and policy-makers should consider revising and updating existing legislation to address emerging concerns in the field of therapeutic digestive enzymes. This may involve conducting comprehensive reviews of regulatory frameworks, considering input from scientific experts, and incorporating provisions that account for technological advancements and new research findings. Pharmaceutical companies should adopt transparent practices in the development, manufacturing, and marketing of therapeutic digestive enzymes. Proactive engagement with regulatory bodies and adherence to established guidelines will be essential. Companies should invest in rigorous testing and research to demonstrate the safety and efficacy of their products, and they should communicate this information clearly to both healthcare professionals and consumers.

## Conclusions

Enzyme supplementation therapy plays a crucial role in the intricate realm of digestive and malabsorption disorders. While animal-derived enzymes continue to be the gold standard, the exploration of plant-based and microbe-derived alternatives signals a transformative era. This evolution is characterized by tailored therapeutic combinations that cater to specific patient needs, expanding beyond enzyme deficiencies to address diverse conditions like CD, thereby presenting a spectrum of potential applications. The complexity introduced by the prospect of combining different enzymes shapes a subtle and tailored future for digestive health.

As this field progresses, a nuanced approach to digestive enzyme supplementation becomes indispensable. The pricing disparities among similar products by different companies in India underscore the need for consumers to stay informed, seek guidance from healthcare professionals, and advocate for responsible marketing practices. Navigating the intricate landscape of digestive enzyme therapy requires adherence to evidence-based practices and a commitment to individualized treatment plans. In conclusion, OE therapy stands at the crossroads of biology, chemistry, and medicine, offering diverse possibilities for therapeutic interventions that are reshaping the treatment landscape in GI healthcare. The synthesis of responsible consumer choices, informed healthcare practices, and ongoing scientific exploration holds the key to shaping a dynamic and promising future for digestive health in the context of the ethical and regulatory challenges faced by Indian pharmaceutical companies.
